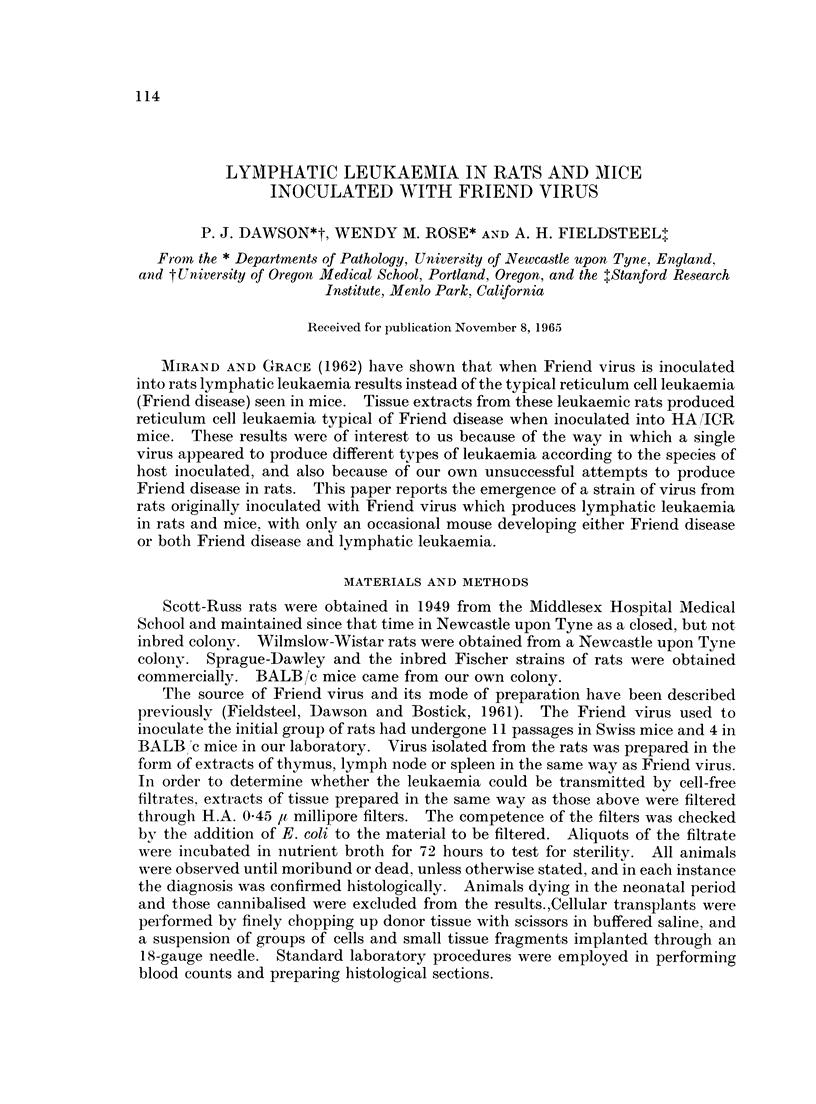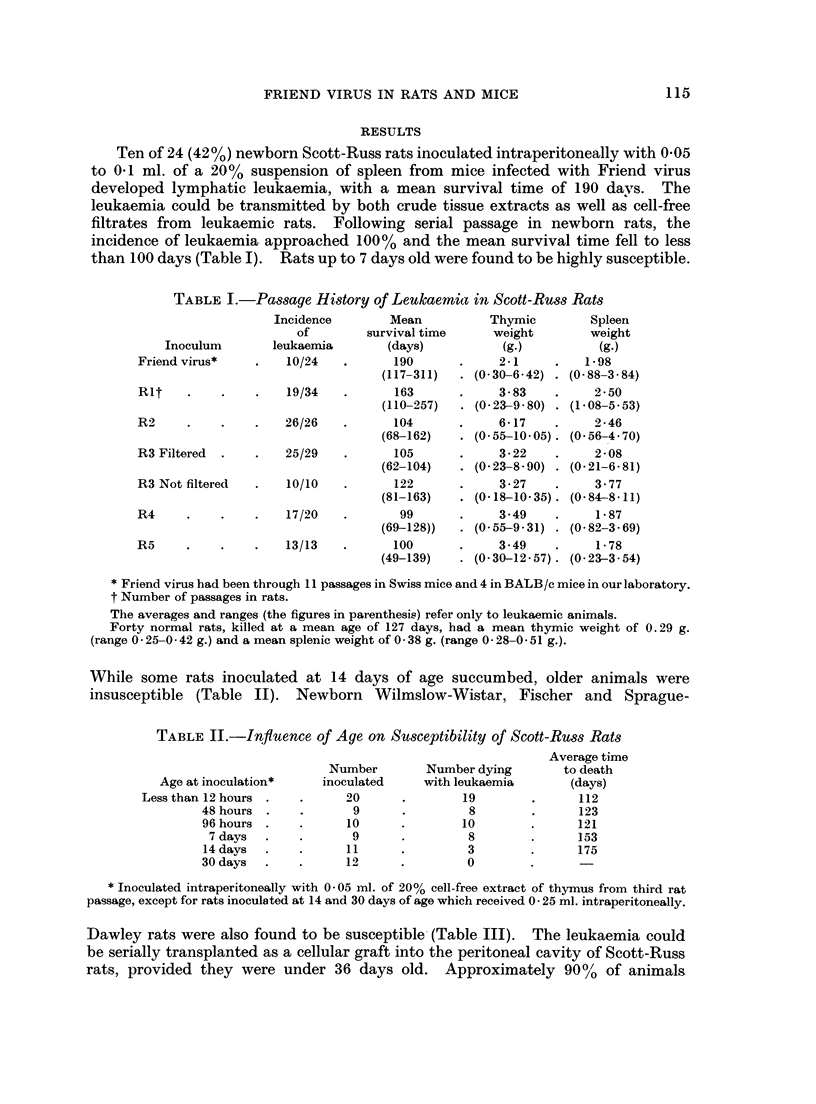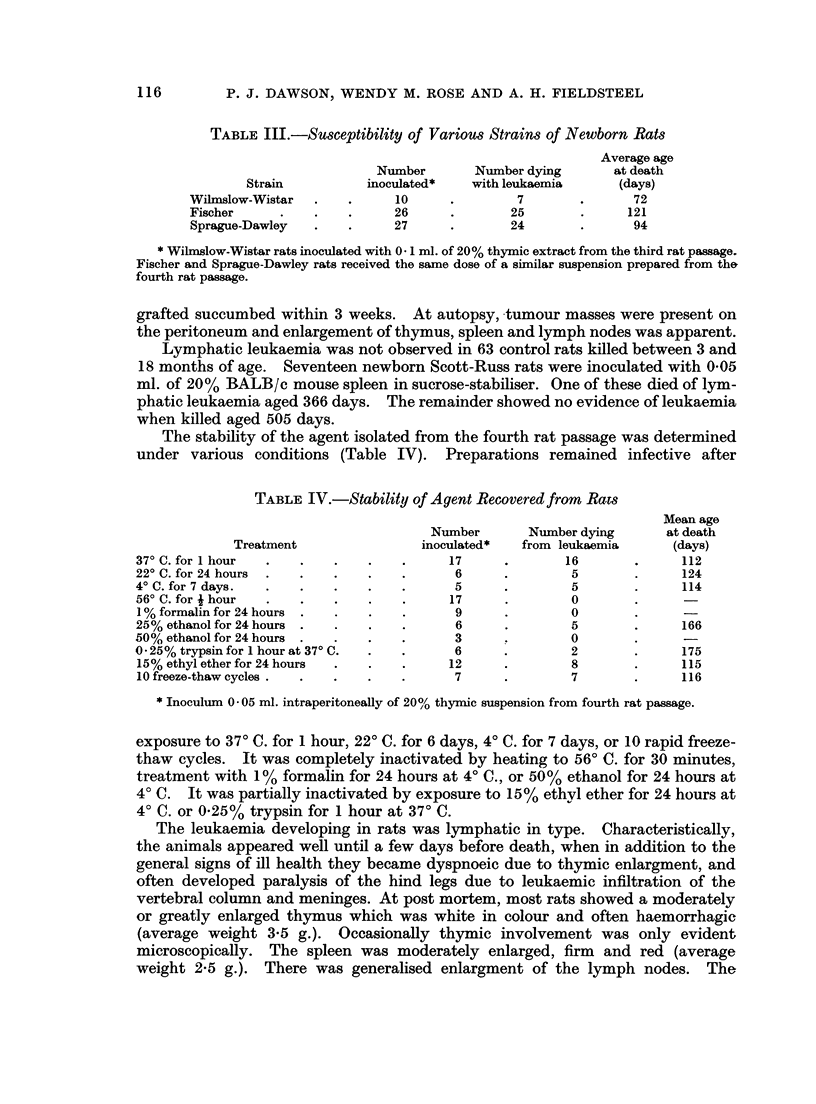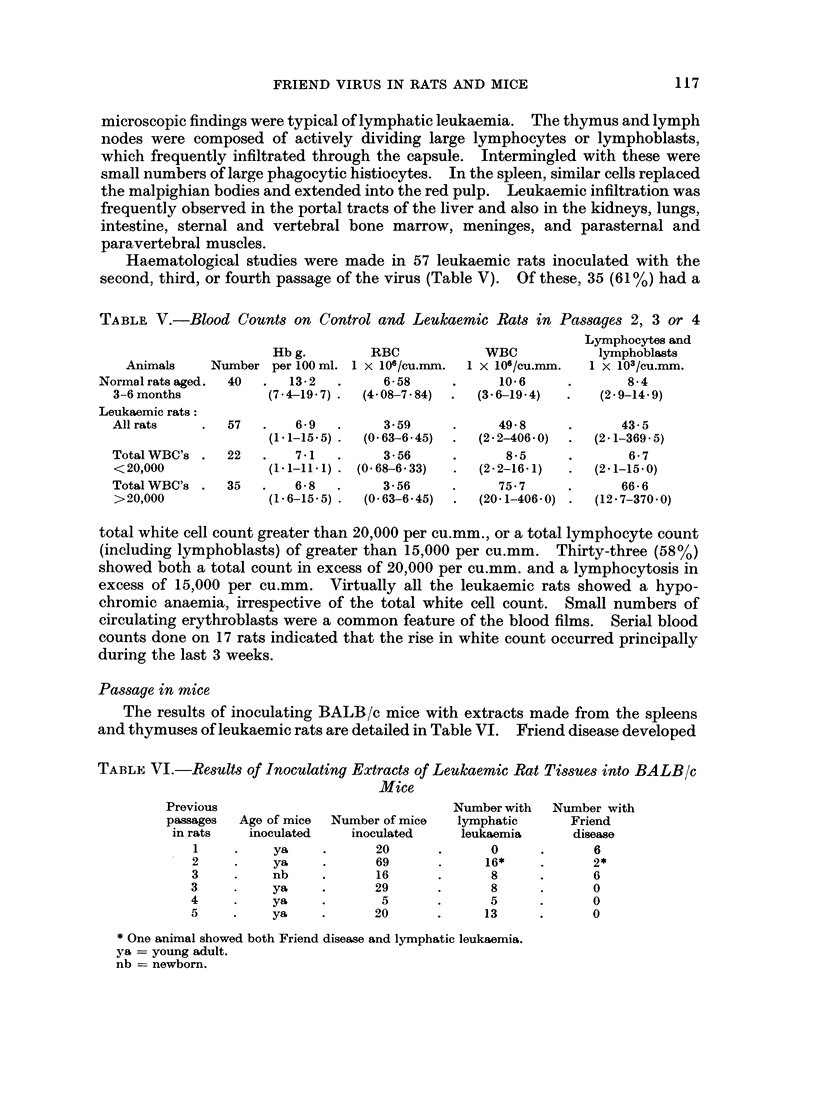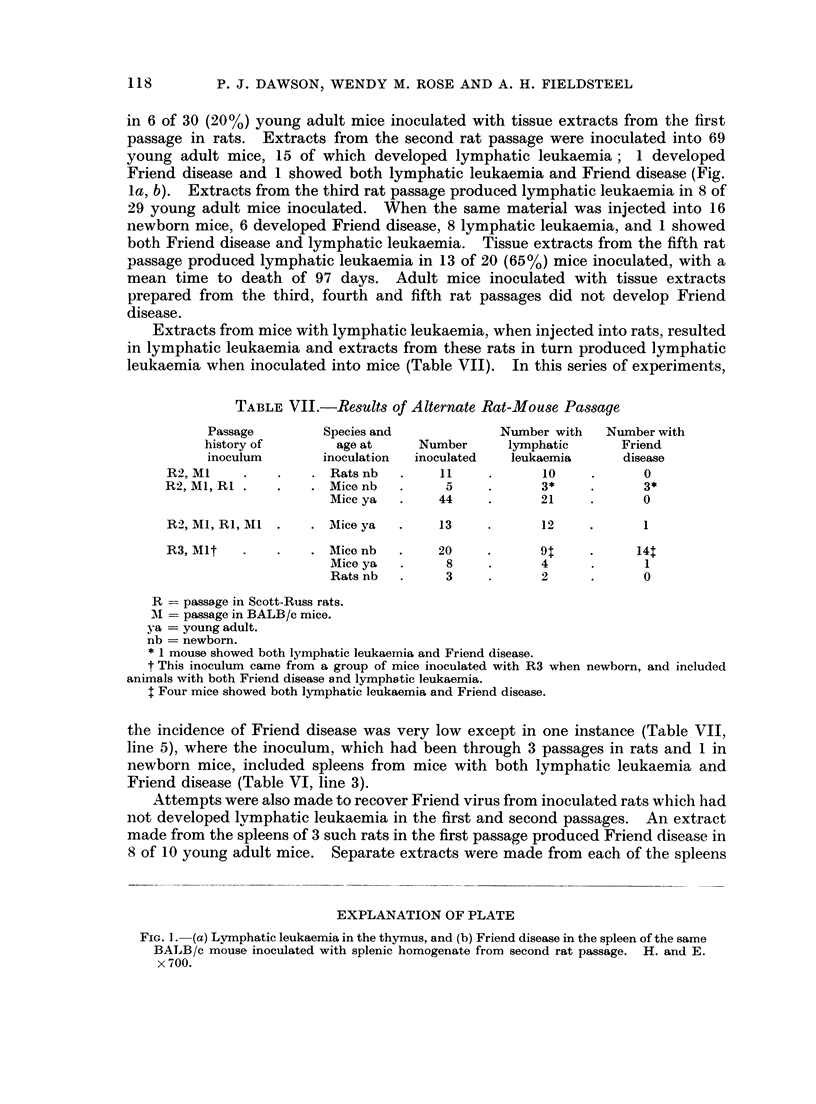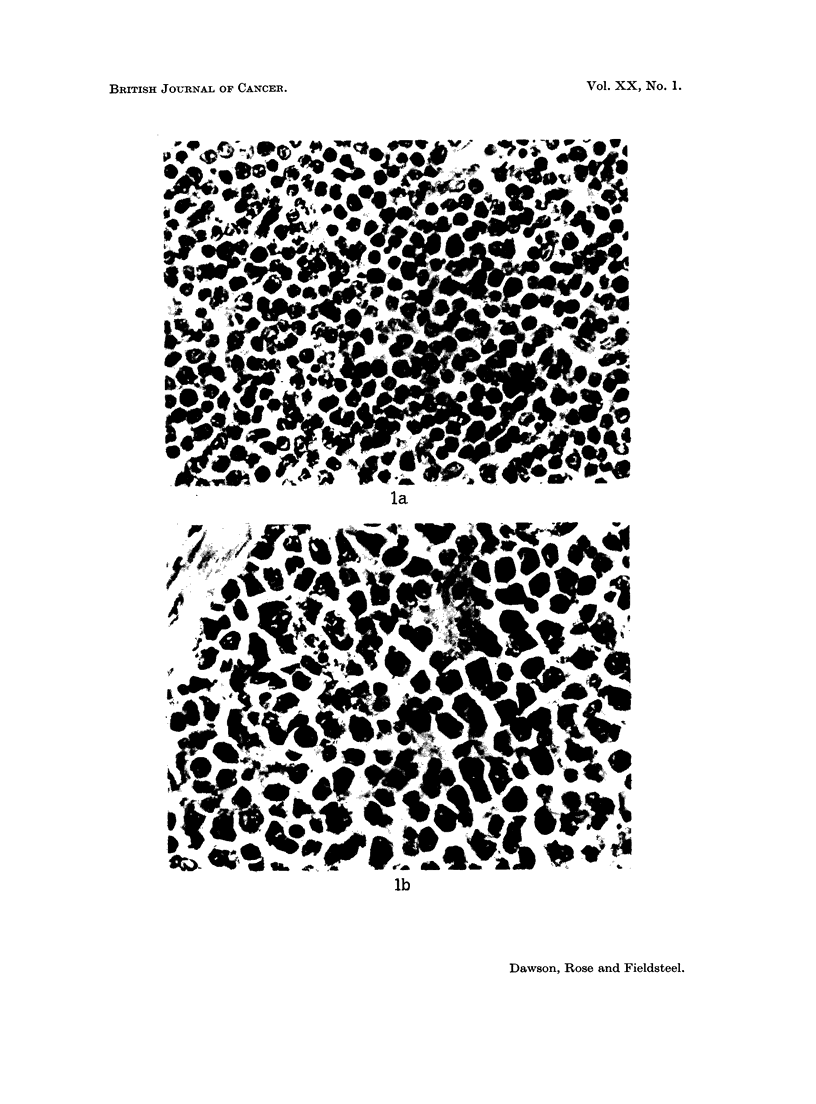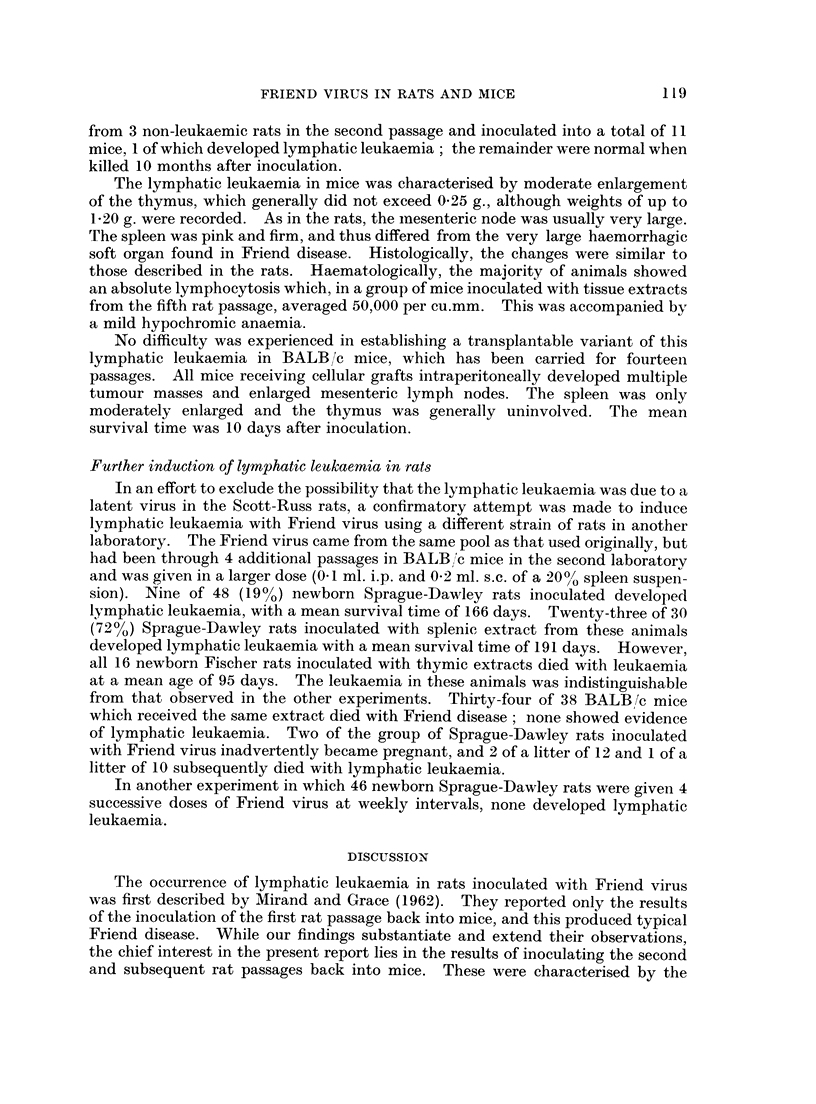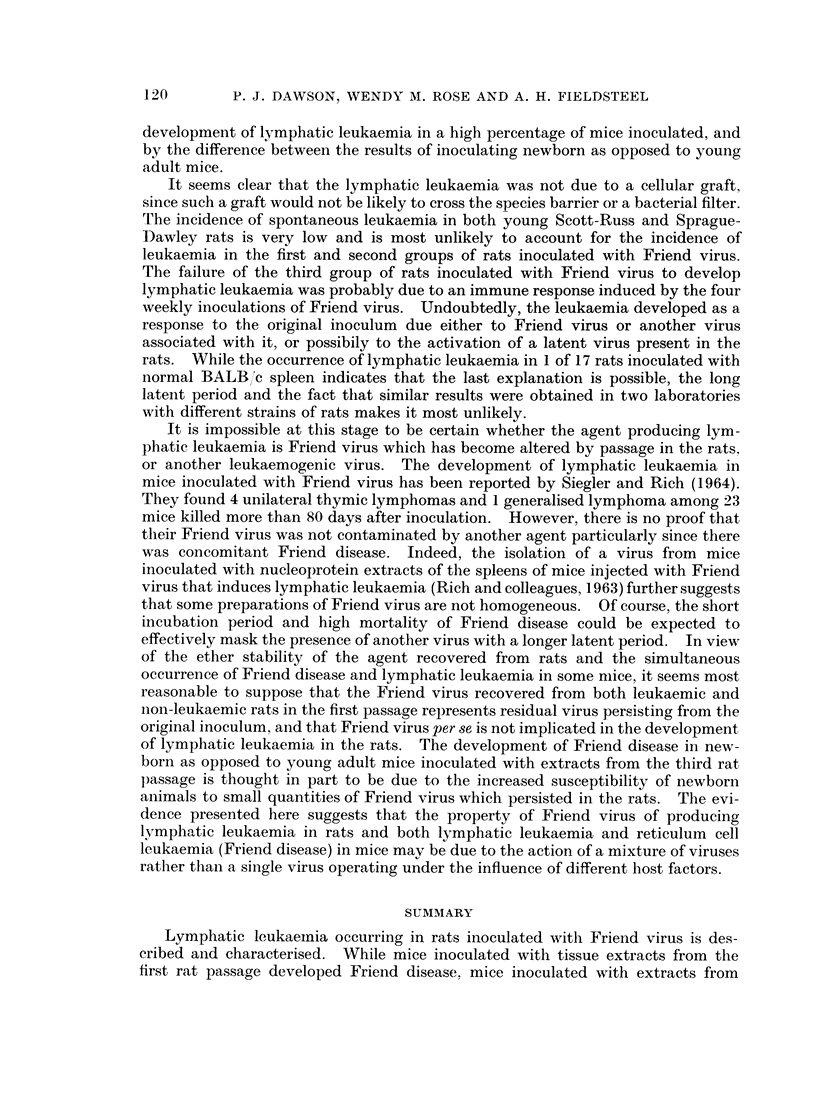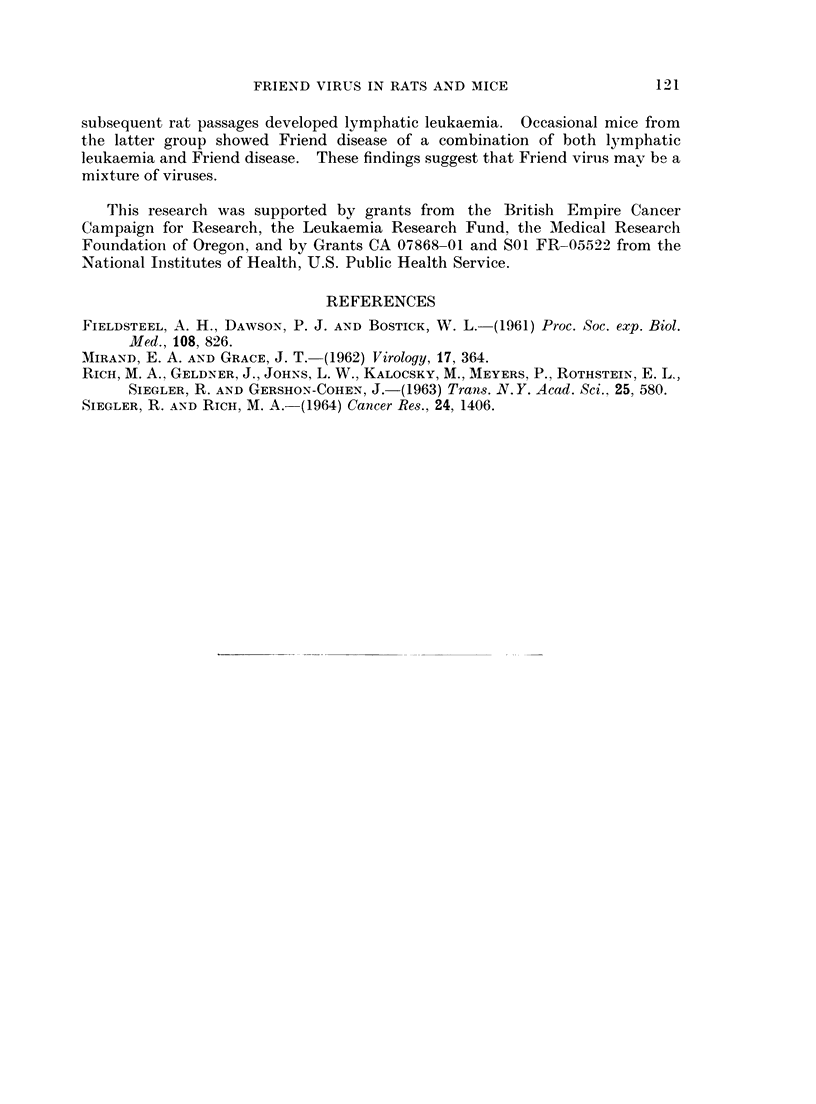# Lymphatic leukaemia in rats and mice inoculated with Friend virus.

**DOI:** 10.1038/bjc.1966.12

**Published:** 1966-03

**Authors:** P. J. Dawson, W. M. Rose, A. H. Fieldsteel

## Abstract

**Images:**


### LYMPHATIC LEUKAEMIA IN RATS AND MICE INOCULATED WITH FRIEND VIRUS

P. J. DAWSON*†, WENDY M. ROSE* AND A. H. FIELDSTEEL‡

*From the * Departments of Pathology, University of Newcastle upon Tyne, England, and †University of Oregon Medical School, Portland, Oregon, and the ‡Stanford Research Institute, Menlo Park, California*

Received for publication November 8, 1965

MIRAND AND GRACE (1962) have shown that when Friend virus is inoculated into rats lymphatic leukaemia results instead of the typical reticulum cell leukaemia (Friend disease) seen in mice. Tissue extracts from these leukaemic rats produced reticulum cell leukaemia typical of Friend disease when inoculated into HA/ICR mice. These results were of interest to us because of the way in which a single virus appeared to produce different types of leukaemia according to the species of host inoculated, and also because of our own unsuccessful attempts to produce Friend disease in rats. This paper reports the emergence of a strain of virus from rats originally inoculated with Friend virus which produces lymphatic leukaemia in rats and mice, with only an occasional mouse developing either Friend disease or both Friend disease and lymphatic leukaemia.

### MATERIALS AND METHODS

Scott-Russ rats were obtained in 1949 from the Middlesex Hospital Medical School and maintained since that time in Newcastle upon Tyne as a closed, but not inbred colony. Wilmslow-Wistar rats were obtained from a Newcastle upon Tyne colony. Sprague-Dawley and the inbred Fischer strains of rats were obtained commercially. BALB/c mice came from our own colony.

The source of Friend virus and its mode of preparation have been described previously (Fieldsteel, Dawson and Bostick, 1961). The Friend virus used to inoculate the initial group of rats had undergone 11 passages in Swiss mice and 4 in BALB/c mice in our laboratory. Virus isolated from the rats was prepared in the form of extracts of thymus, lymph node or spleen in the same way as Friend virus. In order to determine whether the leukaemia could be transmitted by cell-free filtrates, extracts of tissue prepared in the same way as those above were filtered through H.A. 0·45 $\mu$ millipore filters. The competence of the filters was checked by the addition of *E. coli* to the material to be filtered. Aliquots of the filtrate were incubated in nutrient broth for 72 hours to test for sterility. All animals were observed until moribund or dead, unless otherwise stated, and in each instance the diagnosis was confirmed histologically. Animals dying in the neonatal period and those cannibalised were excluded from the results.,Cellular transplants were performed by finely chopping up donor tissue with scissors in buffered saline, and a suspension of groups of cells and small tissue fragments implanted through an 18-gauge needle. Standard laboratory procedures were employed in performing blood counts and preparing histological sections.

### RESULTS

Ten of 24 (42%) newborn Scott-Russ rats inoculated intraperitoneally with 0·05 to 0·1 ml. of a 20% suspension of spleen from mice infected with Friend virus developed lymphatic leukaemia, with a mean survival time of 190 days. The leukaemia could be transmitted by both crude tissue extracts as well as cell-free filtrates from leukaemic rats. Following serial passage in newborn rats, the incidence of leukaemia approached 100% and the mean survival time fell to less than 100 days (Table I). Rats up to 7 days old were found to be highly susceptible.

TABLE I.—*Passage History of Leukaemia in Scott-Russ Rats*

| Inoculum | Incidence of leukaemia | Mean survival time (days) | Thymic weight (g.) | Spleen weight (g.) |
|---|---|---|---|---|
| Friend virus* | 10/24 | 190 (117–311) | 2·1 (0·30–6·42) | 1·98 (0·88–3·84) |
| R1† | 19/34 | 163 (110–257) | 3·83 (0·23–9·80) | 2·50 (1·08–5·53) |
| R2 | 26/26 | 104 (68–162) | 6·17 (0·55–10·05) | 2·46 (0·56–4·70) |
| R3 Filtered | 25/29 | 105 (62–104) | 3·22 (0·23–8·90) | 2·08 (0·21–6·81) |
| R3 Not filtered | 10/10 | 122 (81–163) | 3·27 (0·18–10·35) | 3·77 (0·84–8·11) |
| R4 | 17/20 | 99 (69–128)) | 3·49 (0·55–9·31) | 1·87 (0·82–3·69) |
| R5 | 13/13 | 100 (49–139) | 3·49 (0·30–12·57) | 1·78 (0·23–3·54) |

* Friend virus had been through 11 passages in Swiss mice and 4 in BALB/c mice in our laboratory.
† Number of passages in rats.

The averages and ranges (the figures in parenthesis) refer only to leukaemic animals.
Forty normal rats, killed at a mean age of 127 days, had a mean thymic weight of 0.29 g. (range 0·25–0·42 g.) and a mean splenic weight of 0·38 g. (range 0·28–0·51 g.).

While some rats inoculated at 14 days of age succumbed, older animals were insusceptible (Table II). Newborn Wilmslow-Wistar, Fischer and Sprague-

TABLE II.—*Influence of Age on Susceptibility of Scott-Russ Rats*

| Age at inoculation* | Number inoculated | Number dying with leukaemia | Average time to death (days) |
|---|---|---|---|
| Less than 12 hours | 20 | 19 | 112 |
| 48 hours | 9 | 8 | 123 |
| 96 hours | 10 | 10 | 121 |
| 7 days | 9 | 8 | 153 |
| 14 days | 11 | 3 | 175 |
| 30 days | 12 | 0 | — |

* Inoculated intraperitoneally with 0·05 ml. of 20% cell-free extract of thymus from third rat passage, except for rats inoculated at 14 and 30 days of age which received 0·25 ml. intraperitoneally.

Dawley rats were also found to be susceptible (Table III). The leukaemia could be serially transplanted as a cellular graft into the peritoneal cavity of Scott-Russ rats, provided they were under 36 days old. Approximately 90% of animals

TABLE III.—*Susceptibility of Various Strains of Newborn Rats*

| Strain | Number inoculated* | Number dying with leukaemia | Average age at death (days) |
|---|---|---|---|
| Wilmslow-Wistar | 10 | 7 | 72 |
| Fischer | 26 | 25 | 121 |
| Sprague-Dawley | 27 | 24 | 94 |

* Wilmslow-Wistar rats inoculated with 0·1 ml. of 20% thymic extract from the third rat passage. Fischer and Sprague-Dawley rats received the same dose of a similar suspension prepared from the fourth rat passage.

grafted succumbed within 3 weeks. At autopsy, tumour masses were present on the peritoneum and enlargement of thymus, spleen and lymph nodes was apparent.

Lymphatic leukaemia was not observed in 63 control rats killed between 3 and 18 months of age. Seventeen newborn Scott-Russ rats were inoculated with 0·05 ml. of 20% BALB/c mouse spleen in sucrose-stabiliser. One of these died of lymphatic leukaemia aged 366 days. The remainder showed no evidence of leukaemia when killed aged 505 days.

The stability of the agent isolated from the fourth rat passage was determined under various conditions (Table IV). Preparations remained infective after

TABLE IV.—*Stability of Agent Recovered from Rats*

| Treatment | Number inoculated* | Number dying from leukaemia | Mean age at death (days) |
|---|---|---|---|
| 37° C. for 1 hour | 17 | 16 | 112 |
| 22° C. for 24 hours | 6 | 5 | 124 |
| 4° C. for 7 days | 5 | 5 | 114 |
| 56° C. for ½ hour | 17 | 0 | — |
| 1% formalin for 24 hours | 9 | 0 | — |
| 25% ethanol for 24 hours | 6 | 5 | 166 |
| 50% ethanol for 24 hours | 3 | 0 | — |
| 0·25% trypsin for 1 hour at 37° C. | 6 | 2 | 175 |
| 15% ethyl ether for 24 hours | 12 | 8 | 115 |
| 10 freeze-thaw cycles | 7 | 7 | 116 |

* Inoculum 0·05 ml. intraperitoneally of 20% thymic suspension from fourth rat passage.

exposure to 37° C. for 1 hour, 22° C. for 6 days, 4° C. for 7 days, or 10 rapid freeze-thaw cycles. It was completely inactivated by heating to 56° C. for 30 minutes, treatment with 1% formalin for 24 hours at 4° C., or 50% ethanol for 24 hours at 4° C. It was partially inactivated by exposure to 15% ethyl ether for 24 hours at 4° C. or 0·25% trypsin for 1 hour at 37° C.

The leukaemia developing in rats was lymphatic in type. Characteristically, the animals appeared well until a few days before death, when in addition to the general signs of ill health they became dyspnoeic due to thymic enlargment, and often developed paralysis of the hind legs due to leukaemic infiltration of the vertebral column and meninges. At post mortem, most rats showed a moderately or greatly enlarged thymus which was white in colour and often haemorrhagic (average weight 3·5 g.). Occasionally thymic involvement was only evident microscopically. The spleen was moderately enlarged, firm and red (average weight 2·5 g.). There was generalised enlargment of the lymph nodes. The microscopic findings were typical of lymphatic leukaemia. The thymus and lymph nodes were composed of actively dividing large lymphocytes or lymphoblasts, which frequently infiltrated through the capsule. Intermingled with these were small numbers of large phagocytic histiocytes. In the spleen, similar cells replaced the malpighian bodies and extended into the red pulp. Leukaemic infiltration was frequently observed in the portal tracts of the liver and also in the kidneys, lungs, intestine, sternal and vertebral bone marrow, meninges, and parasternal and paravertebral muscles.

Haematological studies were made in 57 leukaemic rats inoculated with the second, third, or fourth passage of the virus (Table V). Of these, 35 (61%) had a total white cell count greater than 20,000 per cu.mm., or a total lymphocyte count (including lymphoblasts) of greater than 15,000 per cu.mm. Thirty-three (58%) showed both a total count in excess of 20,000 per cu.mm. and a lymphocytosis in excess of 15,000 per cu.mm. Virtually all the leukaemic rats showed a hypochromic anaemia, irrespective of the total white cell count. Small numbers of circulating erythroblasts were a common feature of the blood films. Serial blood counts done on 17 rats indicated that the rise in white count occurred principally during the last 3 weeks.

TABLE V.—*Blood Counts on Control and Leukaemic Rats in Passages* 2, 3 *or* 4

| Animals | Number | Hb g. per 100 ml. | RBC $1 \times 10^6$/cu.mm. | WBC $1 \times 10^6$/cu.mm. | Lymphocytes and lymphoblasts $1 \times 10^3$/cu.mm. |
|---|---|---|---|---|---|
| Normal rats aged 3–6 months | 40 | 13·2 (7·4–19·7) | 6·58 (4·08–7·84) | 10·6 (3·6–19·4) | 8·4 (2·9–14·9) |
| Leukaemic rats: | | | | | |
| All rats | 57 | 6·9 (1·1–15·5) | 3·59 (0·63–6·45) | 49·8 (2·2–406·0) | 43·5 (2·1–369·5) |
| Total WBC's <20,000 | 22 | 7·1 (1·1–11·1) | 3·56 (0·68–6·33) | 8·5 (2·2–16·1) | 6·7 (2·1–15·0) |
| Total WBC's >20,000 | 35 | 6·8 (1·6–15·5) | 3·56 (0·63–6·45) | 75·7 (20·1–406·0) | 66·6 (12·7–370·0) |

*Passage in mice*

The results of inoculating BALB/c mice with extracts made from the spleens and thymuses of leukaemic rats are detailed in Table VI. Friend disease developed

TABLE VI.—*Results of Inoculating Extracts of Leukaemic Rat Tissues into BALB/c Mice*

| Previous passages in rats | Age of mice inoculated | Number of mice inoculated | Number with lymphatic leukaemia | Number with Friend disease |
|---|---|---|---|---|
| 1 | ya | 20 | 0 | 6 |
| 2 | ya | 69 | 16* | 2* |
| 3 | nb | 16 | 8 | 6 |
| 3 | ya | 29 | 8 | 0 |
| 4 | ya | 5 | 5 | 0 |
| 5 | ya | 20 | 13 | 0 |

* One animal showed both Friend disease and lymphatic leukaemia.
ya = young adult.
nb = newborn.

in 6 of 30 (20%) young adult mice inoculated with tissue extracts from the first passage in rats. Extracts from the second rat passage were inoculated into 69 young adult mice, 15 of which developed lymphatic leukaemia ; 1 developed Friend disease and 1 showed both lymphatic leukaemia and Friend disease (Fig. 1*a*, *b*). Extracts from the third rat passage produced lymphatic leukaemia in 8 of 29 young adult mice inoculated. When the same material was injected into 16 newborn mice, 6 developed Friend disease, 8 lymphatic leukaemia, and 1 showed both Friend disease and lymphatic leukaemia. Tissue extracts from the fifth rat passage produced lymphatic leukaemia in 13 of 20 (65%) mice inoculated, with a mean time to death of 97 days. Adult mice inoculated with tissue extracts prepared from the third, fourth and fifth rat passages did not develop Friend disease.

Extracts from mice with lymphatic leukaemia, when injected into rats, resulted in lymphatic leukaemia and extracts from these rats in turn produced lymphatic leukaemia when inoculated into mice (Table VII). In this series of experiments,

TABLE VII.—*Results of Alternate Rat-Mouse Passage*

| Passage history of inoculum | Species and age at inoculation | Number inoculated | Number with lymphatic leukaemia | Number with Friend disease |
|---|---|---|---|---|
| R2, M1 | Rats nb | 11 | 10 | 0 |
| R2, M1, R1 | Mice nb | 5 | 3* | 3* |
| | Mice ya | 44 | 21 | 0 |
| R2, M1, R1, M1 | Mice ya | 13 | 12 | 1 |
| R3, M1† | Mice nb | 20 | 9‡ | 14‡ |
| | Mice ya | 8 | 4 | 1 |
| | Rats nb | 3 | 2 | 0 |

R = passage in Scott-Russ rats.
M = passage in BALB/c mice.
ya = young adult.
nb = newborn.
* 1 mouse showed both lymphatic leukaemia and Friend disease.
† This inoculum came from a group of mice inoculated with R3 when newborn, and included animals with both Friend disease and lymphatic leukaemia.
‡ Four mice showed both lymphatic leukaemia and Friend disease.

the incidence of Friend disease was very low except in one instance (Table VII, line 5), where the inoculum, which had been through 3 passages in rats and 1 in newborn mice, included spleens from mice with both lymphatic leukaemia and Friend disease (Table VI, line 3).

Attempts were also made to recover Friend virus from inoculated rats which had not developed lymphatic leukaemia in the first and second passages. An extract made from the spleens of 3 such rats in the first passage produced Friend disease in 8 of 10 young adult mice. Separate extracts were made from each of the spleens

EXPLANATION OF PLATE

FIG. 1.—(*a*) Lymphatic leukaemia in the thymus, and (b) Friend disease in the spleen of the same BALB/c mouse inoculated with splenic homogenate from second rat passage. H. and E. ×700.

1a

1b

Dawson, Rose and Fieldsteel.

from 3 non-leukaemic rats in the second passage and inoculated into a total of 11 mice, 1 of which developed lymphatic leukaemia ; the remainder were normal when killed 10 months after inoculation.

The lymphatic leukaemia in mice was characterised by moderate enlargement of the thymus, which generally did not exceed 0·25 g., although weights of up to 1·20 g. were recorded. As in the rats, the mesenteric node was usually very large. The spleen was pink and firm, and thus differed from the very large haemorrhagic soft organ found in Friend disease. Histologically, the changes were similar to those described in the rats. Haematologically, the majority of animals showed an absolute lymphocytosis which, in a group of mice inoculated with tissue extracts from the fifth rat passage, averaged 50,000 per cu.mm. This was accompanied by a mild hypochromic anaemia.

No difficulty was experienced in establishing a transplantable variant of this lymphatic leukaemia in BALB/c mice, which has been carried for fourteen passages. All mice receiving cellular grafts intraperitoneally developed multiple tumour masses and enlarged mesenteric lymph nodes. The spleen was only moderately enlarged and the thymus was generally uninvolved. The mean survival time was 10 days after inoculation.

*Further induction of lymphatic leukaemia in rats*

In an effort to exclude the possibility that the lymphatic leukaemia was due to a latent virus in the Scott-Russ rats, a confirmatory attempt was made to induce lymphatic leukaemia with Friend virus using a different strain of rats in another laboratory. The Friend virus came from the same pool as that used originally, but had been through 4 additional passages in BALB/c mice in the second laboratory and was given in a larger dose (0·1 ml. i.p. and 0·2 ml. s.c. of a 20% spleen suspension). Nine of 48 (19%) newborn Sprague-Dawley rats inoculated developed lymphatic leukaemia, with a mean survival time of 166 days. Twenty-three of 30 (72%) Sprague-Dawley rats inoculated with splenic extract from these animals developed lymphatic leukaemia with a mean survival time of 191 days. However, all 16 newborn Fischer rats inoculated with thymic extracts died with leukaemia at a mean age of 95 days. The leukaemia in these animals was indistinguishable from that observed in the other experiments. Thirty-four of 38 BALB/c mice which received the same extract died with Friend disease ; none showed evidence of lymphatic leukaemia. Two of the group of Sprague-Dawley rats inoculated with Friend virus inadvertently became pregnant, and 2 of a litter of 12 and 1 of a litter of 10 subsequently died with lymphatic leukaemia.

In another experiment in which 46 newborn Sprague-Dawley rats were given 4 successive doses of Friend virus at weekly intervals, none developed lymphatic leukaemia.

### DISCUSSION

The occurrence of lymphatic leukaemia in rats inoculated with Friend virus was first described by Mirand and Grace (1962). They reported only the results of the inoculation of the first rat passage back into mice, and this produced typical Friend disease. While our findings substantiate and extend their observations, the chief interest in the present report lies in the results of inoculating the second and subsequent rat passages back into mice. These were characterised by the development of lymphatic leukaemia in a high percentage of mice inoculated, and by the difference between the results of inoculating newborn as opposed to young adult mice.

It seems clear that the lymphatic leukaemia was not due to a cellular graft, since such a graft would not be likely to cross the species barrier or a bacterial filter. The incidence of spontaneous leukaemia in both young Scott-Russ and Sprague-Dawley rats is very low and is most unlikely to account for the incidence of leukaemia in the first and second groups of rats inoculated with Friend virus. The failure of the third group of rats inoculated with Friend virus to develop lymphatic leukaemia was probably due to an immune response induced by the four weekly inoculations of Friend virus. Undoubtedly, the leukaemia developed as a response to the original inoculum due either to Friend virus or another virus associated with it, or possibily to the activation of a latent virus present in the rats. While the occurrence of lymphatic leukaemia in 1 of 17 rats inoculated with normal BALB/c spleen indicates that the last explanation is possible, the long latent period and the fact that similar results were obtained in two laboratories with different strains of rats makes it most unlikely.

It is impossible at this stage to be certain whether the agent producing lymphatic leukaemia is Friend virus which has become altered by passage in the rats, or another leukaemogenic virus. The development of lymphatic leukaemia in mice inoculated with Friend virus has been reported by Siegler and Rich (1964). They found 4 unilateral thymic lymphomas and 1 generalised lymphoma among 23 mice killed more than 80 days after inoculation. However, there is no proof that their Friend virus was not contaminated by another agent particularly since there was concomitant Friend disease. Indeed, the isolation of a virus from mice inoculated with nucleoprotein extracts of the spleens of mice injected with Friend virus that induces lymphatic leukaemia (Rich and colleagues, 1963) further suggests that some preparations of Friend virus are not homogeneous. Of course, the short incubation period and high mortality of Friend disease could be expected to effectively mask the presence of another virus with a longer latent period. In view of the ether stability of the agent recovered from rats and the simultaneous occurrence of Friend disease and lymphatic leukaemia in some mice, it seems most reasonable to suppose that the Friend virus recovered from both leukaemic and non-leukaemic rats in the first passage represents residual virus persisting from the original inoculum, and that Friend virus *per se* is not implicated in the development of lymphatic leukaemia in the rats. The development of Friend disease in newborn as opposed to young adult mice inoculated with extracts from the third rat passage is thought in part to be due to the increased susceptibility of newborn animals to small quantities of Friend virus which persisted in the rats. The evidence presented here suggests that the property of Friend virus of producing lymphatic leukaemia in rats and both lymphatic leukaemia and reticulum cell leukaemia (Friend disease) in mice may be due to the action of a mixture of viruses rather than a single virus operating under the influence of different host factors.

### SUMMARY

Lymphatic leukaemia occurring in rats inoculated with Friend virus is described and characterised. While mice inoculated with tissue extracts from the first rat passage developed Friend disease, mice inoculated with extracts from subsequent rat passages developed lymphatic leukaemia. Occasional mice from the latter group showed Friend disease of a combination of both lymphatic leukaemia and Friend disease. These findings suggest that Friend virus may be a mixture of viruses.

This research was supported by grants from the British Empire Cancer Campaign for Research, the Leukaemia Research Fund, the Medical Research Foundation of Oregon, and by Grants CA 07868–01 and S01 FR–05522 from the National Institutes of Health, U.S. Public Health Service.

### REFERENCES

Fieldsteel, A. H., Dawson, P. J. and Bostick, W. L.—(1961) *Proc. Soc. exp. Biol. Med.*, **108**, 826.

Mirand, E. A. and Grace, J. T.—(1962) *Virology*, **17**, 364.

Rich, M. A., Geldner, J., Johns, L. W., Kalocsky, M., Meyers, P., Rothstein, E. L., Siegler, R. and Gershon-Cohen, J.—(1963) *Trans. N.Y. Acad. Sci.*, **25**, 580.

Siegler, R. and Rich, M. A.—(1964) *Cancer Res.*, **24**, 1406.